# Inflammation but Not Dietary Macronutrients Insufficiency Associated with the Malnutrition-Inflammation Score in Hemodialysis Population

**DOI:** 10.1371/journal.pone.0083233

**Published:** 2013-12-11

**Authors:** Jie Chen, Hongquan Peng, Long Xiao, Kun Zhang, Zhimin Yuan, Jianping Chen, Zhiyu Wang, Jingfeng Wang, Hui Huang

**Affiliations:** 1 Guangdong Province Key Laboratory of Arrhythmia and Electrophysiology, Guangzhou, Guangdong Province, China; 2 Department of Cardiology, Sun Yat-sen Memorial Hospital of Sun Yat-sen University, Guangzhou, Guangdong Province, China; 3 Department of Radiation Oncology, Sun Yat-sen Memorial Hospital of Sun Yat-sen University, Guangzhou, Guangdong Province, China; 4 Renal Division, Kiang Wu Hospital, Macau SAR, China; 5 Department of blood purification of the Second Affiliated Hospital of Guangzhou Medical College, Guangzhou, Guangdong Province, China; 6 Department of Nutrition, Sun Yat-sen Memorial Hospital of Sun Yat-sen University, Guangzhou, Guangdong Province, China; 7 School of Chinese Medicine, The University of Hong Kong, Hong Kong, China; Wageningen University, Netherlands

## Abstract

Malnutrition is associated with increased risk of mortality in hemodialysis patients. And insufficient dietary intake is the common cause for malnutrition. So, in order to survey the dietary intake of hemodialysis patients and study the relationship between the dietary feature and nutritional status, a cross-sectional study was performed. 75 hemodialysis patients from South China participated in the dietary intake survey and nutrition assessment. A three-day diet diary record was used to estimate the major dietary macronutrients. Nutritional status was assessed by malnutrition-inflammation score (MIS) in addition to several related anthropometric measurements. Serum albumin, transferrin, and high-sensitivity C-reactive protein (CRP) were measured. Receiver operating characteristic (ROC) curve analysis was used to quantify the assessing value of independent parameters for nutritional status. The results showed that 48% patients were malnourished according to the MIS. The malnourished patients had a lower body mass index (BMI), fat mass (FM), albumin and a higher level of CRP, compared with normal nourished patients (*P* < 0.05). However, no significant differences of macronutrients (calories, protein, fat, carbohydrates, etc) were found between the two nutrition groups (*P* > 0.05). The multivariate regression analysis showed that the major macronutrients had no significant association with MIS (*P* > 0.05). In conclusion, malnutrition is very common in South China hemodialysis population and these data indicated that inflammation but not dietary macronutrients insufficiency might be the candidate cause for malnutrition in hemodialysis population.

## Introduction

Assessment of nutritional status is imperative for health promotion and disease prevention in hemodialysis population. As well known, malnutrition is closely associated with decreased quality of life, increased risk of mortality in hemodialysis patients [1-3]. And early evaluation of nutritional status will be helpful to optimize the clinical management of hemodialysis patients [4-6]. Insufficient dietary intake is one of the most common and important reasons for malnutrition in dialysis patients [7]. Hence, dietary intake assessment is of particular importance for evaluating malnutrition in hemodialysis patients. Generally, analyzing the quality and quantity of various types of dietary macronutrients is a common way to assess the dietary intake of hemodialysis patients. However, currently the assessment of nutritional status and dietary intake were often neglected in Chinese hemodialysis patients. With the economic development, meat, chicken, fish and milk, which are scarce in the past, are produced in large quantities. While the mortality of hemodialysis patients is still in a high level.

Nutritional status assessment can be reached by some ways, such as body mass index (BMI), subjective global assessment (SGA), malnutrition-inflammation score (MIS) and dietary intake assessment. SGA was firstly designed by Detsky et al. [8]. In comparison with SGA, MIS scoring system emphasizes the effect of inflammation, and is another comprehensive system measuring nutritional and inflammatory status of hemodialysis patients. And MIS is correlated with morbidity and mortality in maintenance hemodialysis patients [9,10]. However, it has not been routinely used in Chinese population [11]. Moreover, there exist different demographic characteristics and different dietary patterns of Chinese population compared to westerners. 

So, in the current study, we assessed the nutritional status of hemodialysis patients by MIS score system and analyzed the association between MIS and anthropometric measurements so as to determine which nutritional markers are better predictors for assessment of nutritional status. In addition, whether malnutrition in Chinese hemodialysis patients is associated with dietary macronutrients intake are still unknown. Therefore, in this study, dietary macronutrients intake of hemodialysis patients were also investigated. We examined the correlation of dietary macronutrients intake with MIS in hemodialysis patients to determine whether dietary macronutrients were associated with malnutrition and independently influenced malnutrition. 

## Methods

### Ethical statement

This study protocol conformed to the ethical guidelines of the 1975 Declaration of Helsinki as reflected in a priori approval by the Ethics Committee of Sun Yat-sen University. Patients enrolled in this study were from 4 teaching hospitals in South China (Sun Yat-sen Memorial Hospital; the Second Affiliated Hospital of Guangzhou Medical College; Kiang Wu Hospital; Affiliated hospital of School of Chinese Medicine, The University of Hong Kong). Written Informed consent was obtained from each participant and their medical records were studied by anonymous means. 

### Study population

**Figure 1:**
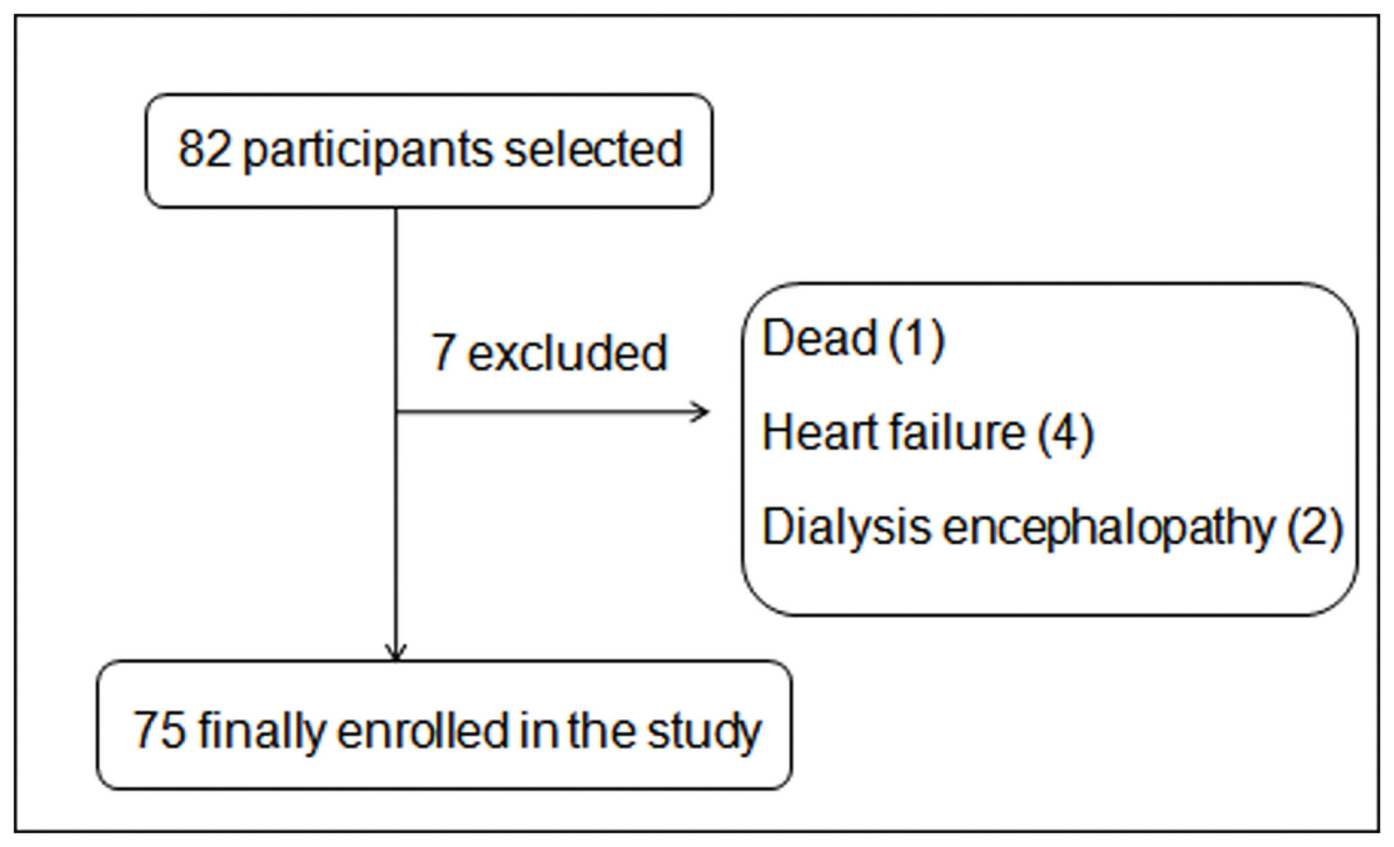
A flow chart of selection.

This was a cross-sectional, descriptive-analytic study and seventy-five randomly selected maintenance hemodialysis patients participated in the dietary and nutritional assessment (a flow chart of selection, Figure 1). The patients with acute renal failure, major adverse cardiovascular events, cancer, severe gastrointestinal and hepatic diseases, and severe infections were excluded. Clinical data, such as patients’ demographics and duration of hemodialysis, were obtained by a detailed history elucidation from the patients and their cases records. 

### Anthropometric measurements

Several anthropometric measurements were done after termination of the hemodialysis sessions. The skin fold thickness of triceps, biceps, subscapular and superior iliac estimated the subcutaneous fat deposits. They were measured using skin fold caliper. Density (D) was first calculated from the sum of the skinfold thicknesses by using the hypothesis that this sum value is the most informative [12]. Next, the percentage of fat mass (FM) was calculated according to the Siri equation: FM (%)=100×(4.95/D-4.5) [13]. Body dry weight was measured between 10 and 20 minutes after termination of the hemodialysis sessions. All measurements were performed three times on the non-access arms. The BMI was calculated as the ratio between end-hemodialysis body weight in kg and the square of height in meters (kg/m^2^).

### Analysis of three-day dietary

A three-day diet diary record assessed by a well-trained dietitian was used to estimate the daily dietary intake [9]. Dietary intake was recalled over the last hemodialysis treatment day of the week and the two subsequent non-dialysis days. The dietitians made changes and corrections on the food record and used the Minnesota Nutrient Data System software (version 2005; Nutrition Coordinating Center, Minneapolis, Minn, USA) to complete the nutrient analysis [14]. The Dietwin® nutrition software (version 8.0) was used for the dietary quantification of the food recalls. To determine dietary intake, patients were required to record the amount of each food and the daily number of meals (breakfast, lunch, dinner and snacks, etc.) in their dietary diaries. Skilled dieticians trained patients how to record the total food intake in the diary by household measures, and also instructed them how to take the measures of the utensils before starting food record.

### Laboratory test

 During this study, the values of routine laboratory data were performed every 3-month. Pre-hemodialysis blood samples were obtained. The measurement of serum albumin, serum transferrin and high-sensitivity C-reactive protein (CRP) were tested. Serum albumin level was determined by a bromocresol green dye binding colorimetric assay. Serum transferrin level was measured by the Ciba-Cornings Automated Chemiluminescence System (ACS180), employing a two-site chemiluminometric (sandwich) immunoassay, which uses constant amounts of two anti-ferritin antibodies. Serum CRP level was measured with a high sensitive latex-enhanced immunoturbidimetric assay (Randox laboratory Ltd, Belfast, United Kingdom).

### Nutritional evaluation Scores

Nutritional scoring assessed by MIS system was performed among all the participating hemodialysis patients. MIS has 10 components: weight change, dietary intake, gastrointestinal symptoms, functional capacity, comorbidity, subcutaneous fat, muscle wasting, BMI, serum albumin level, and total iron-binding capacity or serum transferrin level. The ten items of MIS were scored independently. Each item was scored by four different levels from 0 (normal) to 3 (very severe). The total scores of 10 items range from 0 to 30, indicating the severity degree of malnutrition and inflammation [9]. 

### Statistical analysis

Descriptive statistics and regression analysis were carried out with the statistical software package (SPSS17.0). All normally distributed data were expressed as mean ± SD. Before making comparison between groups, Shapiro-Wilk test for normality was tested. Differences of normal distributed continuous variables between groups were determined by unpaired t-test, while non-normal distributed continuous variables were compared by Mann-Whitney U-test. Multivariate regression analysis was used to assess the strength of association between MIS and dietary intake /anthropometric parameters and evaluate whether macronutrients were independent factors influencing malnutrion. Receiver operating characteristic (ROC) curve analysis was used to quantify the assessing value of independent parameters for nutritional status. For all statistical tests, a P-value of < 0.05 was considered statistically significant. 

## Results

### Demographic characteristics between two nutrition groups in hemodialysis patients

A total of 75 patients (male/female: 51/24) undergoing maintenance hemodialysis were recruited. The mean duration was 3.29 ± 1.08 years. According to the MIS, we grouped the whole hemodialysis patients into two groups: normal nutrition group and malnutrition group. The baseline demographic characteristics were showed in Table 1. The data showed that BMI, FM, and serum albumin decreased significantly in malnutrition group (BMI, *P* = 0.013; FM, *P* = 0.039; albumin, *P* = 0.037), while CRP and MIS significantly increased (CRP, *P* = 0.008; MIS, *P* = 0.000). However, no significance was found in age, height, body dry weight, and transferrin (*P* > 0.05, Table 1). 

**Table 1:** Demographic characteristics between two nutrition groups in hemodialysis patients.

Variable	normal	malnutrition	P value
	n=39	n=36	
Age (year)	63±11.9	63±14.0	0.36
Height (m)	1.6±0.06	1.6±0.07	0.47
BDW (kg)	60±8.4	56±9.4	0.073
BMI (kg/m^2^)	22.3±2.6	20.5 ±3.4	0.013*
Transferrin (ug/L)	41.3±23.3	40.0±18.1	0.90
Albumin (g/L)	41±4.3	37±5.0	0.037*
FM (kg)	15±5.1	12±5.4	0.039*
CRP (mg/L)	1.5±0.8	4.4±2.4	0.008*
MIS	8±2.2	15±3.4	0.000*

All values are expressed as mean±SD.

P-value based on t-test for parametric continuous variables is for comparison between the two groups: normal nutrition group and malnutrition group (*P <0.05).

Abbreviations: BDW, body dry weight; BMI, body mass index; FM, fat mass; CRP, high-sensitivity C-reactive protein; MIS, malnutrition-inflammation score.

### Comparison of dietary macronutrients in different nutrition groups

The accurate dietary intake of the nutrients was described in Table 2. No significant differences of energy, protein, fat, carbohydrates, dietary fiber, and cholesterol intake were found between the normal nutrition group and malnutrition group (*P* > 0.05) (Table 2). 

**Table 2:** Comparison of dietary macronutrients in different nutrition groups according to MIS in hemodialysis patients.

Variable	normal	malnutrition	P value
	n=39	n=36	
Energy(kcal)	1891±387.0	1772±395.8	0.22
-kcal/Kg.d	32.5±7.6	31.9±6.3	0.70
Protein(g)	79±19.2	71±19.7	0.11
-g/kg.d	1.3±0.3	1.3±0.4	0.85
-Protein%	17±2.9	16±2.4	0.23
Fat(g)	65±17.0	62 ±16.8	0.34
-g/kg.d	1.1±0.3	1.1±0.4	0.79
-Fat%	31±6.3	33±6.4	0.35
Carbohydrates (g)	250±60.9	223±61.4	0.091
-Kg.d	4.2±1.03	4.1±1.10	0.67
-carbohydrate%	53±8.2	52±6.7	0.50
Dietary fiber (g)	10±4.6	8±3.4	0.081
Cholesterol(mg)	515±288.6	441±225.2	0.42

All values are expressed as mean±SD.

P-value based on t-test for parametric continuous variables is for comparison among the two groups: normal nutrition and malnutrition (*P <0.05).

### The correlation analysis among MIS and anthropometric measurements, dietary macronutrients and CRP

Then we try to confirm the above findings through a multivariate regression analysis (see Table 3). It was found that both BMI and FM were associated with MIS independent of dietary intake after adjustment of age, sex and height (BMI, B = -4.54, *P* = 0.0005; FM, B = -4.67, *P* = 0.0003). And it is particularly concerned to note that the levels of CRP also had a significant association with MIS (B = -0.56; *P* = 0.0001) while the major dietary macronutrients did not show significant association with MIS (*P* > 0.05). 

**Table 3:** The multivariate regression analysis among MIS and anthropometric measurements, dietary macronutrients, and CRP.

Variable	MIS	
	B	P value
Age (year)	-0.051	0.68
Sex	0.013	0.71
Height (m)	0.011	0.78
BMI (kg/m^2^)	-4.54	0.0005*
FM (kg)	-4.67	0.0003*
Protein (g)	0.041	0.23
Fat (g)	0.015	0.67
Carbohydrates (g)	-0.004	0.21
Dietary fiber (g)	0.063	0.62
Cholesterol (mg)	-0.003	0.11
CRP (mg/L)	-0.56	0.0001*

P-value based on the multivariate regression analysis (*P < 0.05).

Abbreviations: BMI, body mass index; CRP, high-sensitivity C-reactive protein; FM, fat mass; MIS, malnutrition-inflammation score.

### Markers for assessing nutritional status

**Figure 2:**
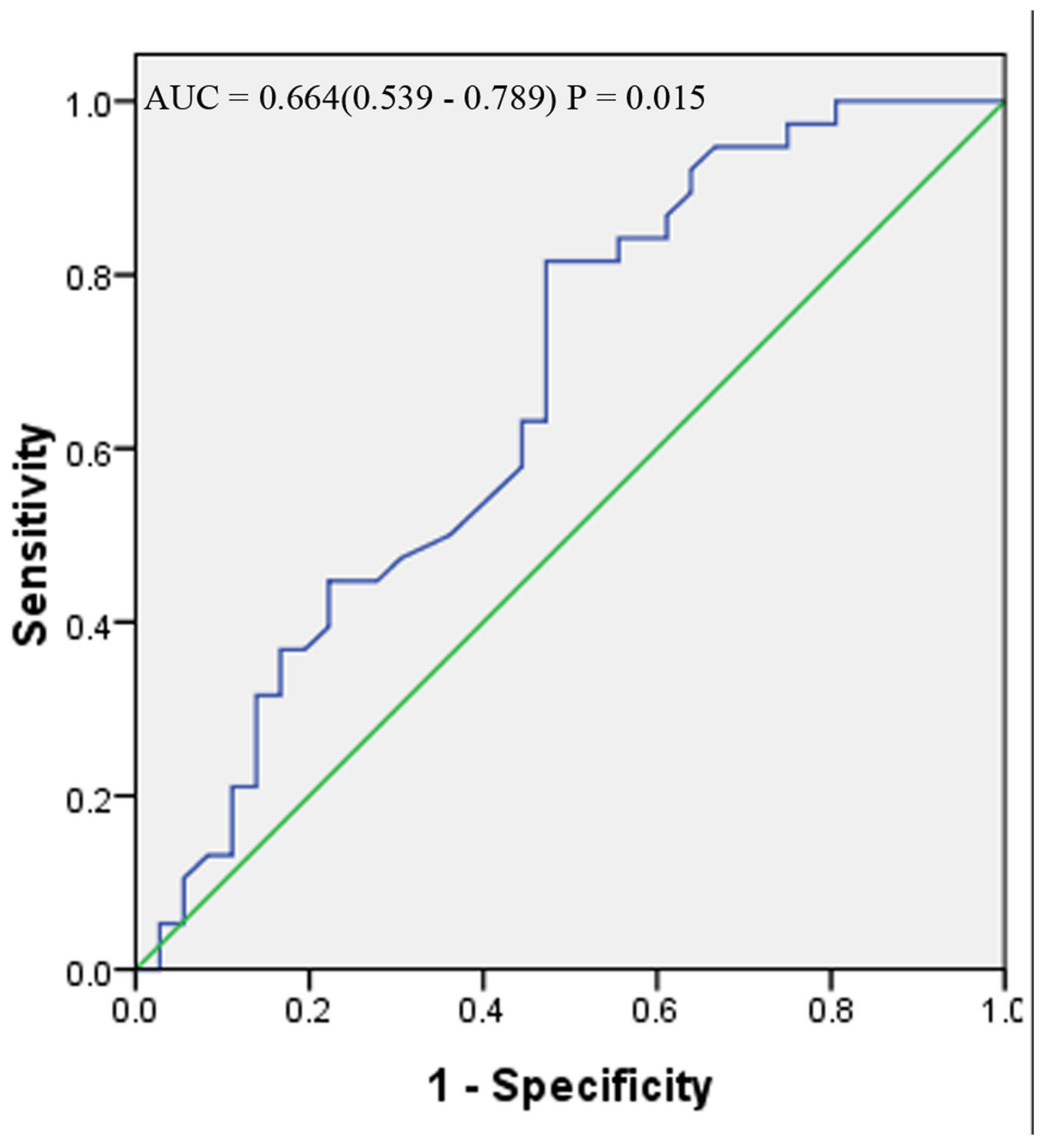
Receiver-operating characteristic (ROC) curve for anthropometric assessment BMI in assessing nutrition status using MIS as the reference standard (*P < 0.05). For each screening test, sensitivity is plotted against 100-specificity.

**Figure 3:**
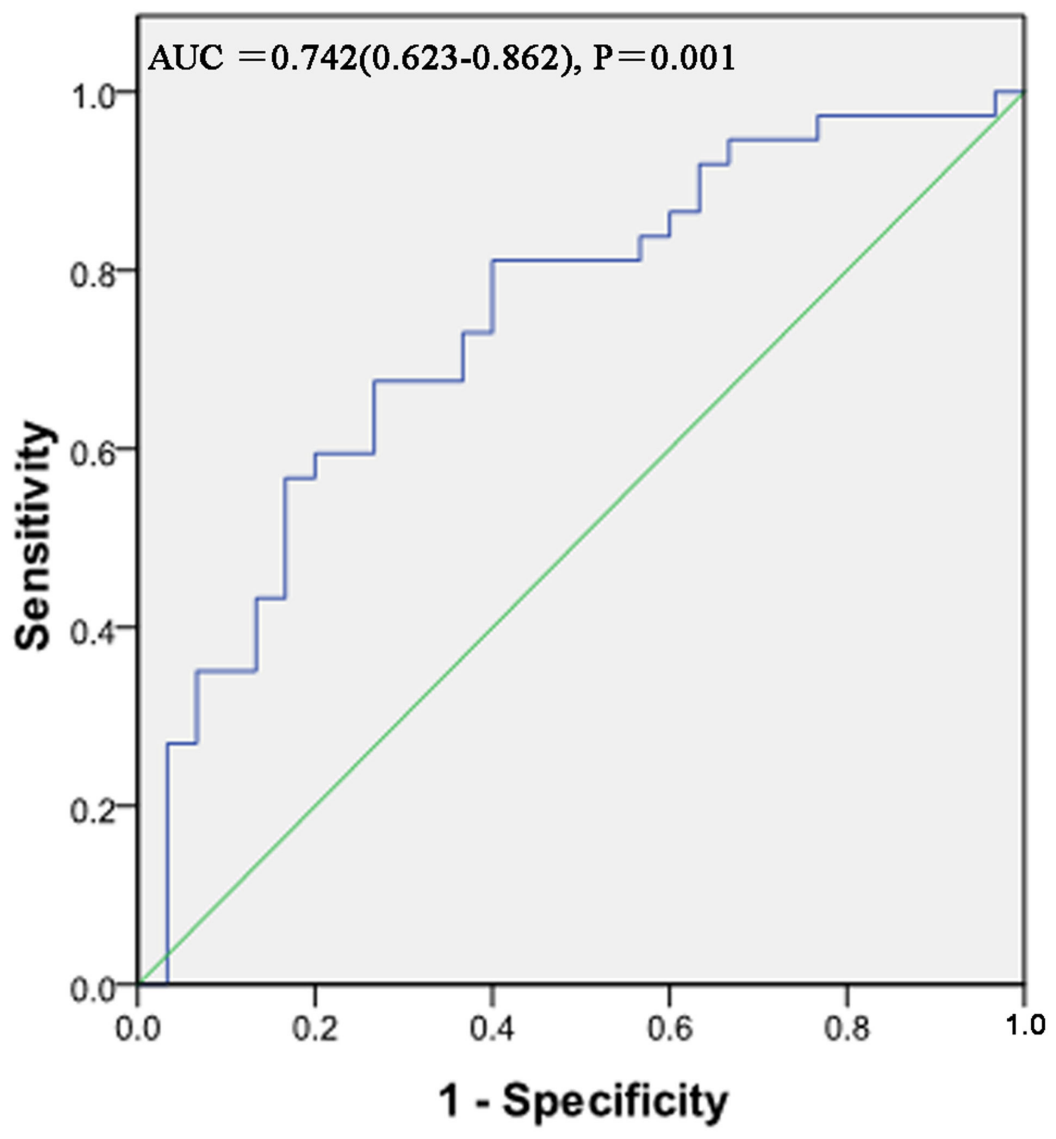
Receiver-operating characteristic (ROC) curve for anthropometric assessment FM in assessing nutrition status using MIS as the reference standard (*P < 0.05). For each screening test, sensitivity is plotted against 100-specificity.

The ROC curve analysis was further used to analyze whether BMI and FM could be better predictors for nutritional status evaluation using MIS as the reference standard. The area under the curve (AUC) of BMI was 0.664 (0.539-0.789) (*P* = 0.015). And BMI provided 84.2% sensitivity and 44.4% specificity with a threshold value of 19.5 g/m^2^ (Figure 2). The AUC of FM was 0.742 (0.623-0.862) (*P* = 0.001). FM provided 81.1% sensitivity and 60.0% specificity with a threshold value of 12.24 cm (Figure 3). 

## Discussion

Malnutrition is extremely common in hemodialysis patients. Malnutrition is defined as a consequence of long-term protein-energy imbalance and changes in dietary intake. Malnutrition can be assessed by different methods. However, the best and earliest markers to define patients at a high risk of malnutrition are still needed to be determined. Since malnutrition is not identified by only one or two markers, it may be advisable to define malnutrition by multiple markers in a broader spectrum. Currently, the nutritional status evaluation is often ignored and the assessment strategy is not definite in hemodialysis population in China. Although some markers e.g. body weight, serum albumin, etc are effective in identifying the patients with high risks of malnutrition, they have some limitations and can be influenced by other non-nutritional factors [15,16]. 

MIS is a reproducible and relative standard method for assessing nutrition and inflammation of hemodialysis patients, and is correlated with morbidity and mortality in maintenance hemodialysis patients [9]. However, it is not widely used in China. Our present study showed that FM and BMI were good and convenient nutritional markers associated with MIS. As we all know, BMI is a simple and an objective measurement to determine nutritional status, but it can be influenced by edema or serous cavity effusion [17,18]. And the ROC curve analysis indicated that FM had a higher specificity than BMI which indicated that FM was a relatively better and valid marker for assessing nutritional status. 

Nutrient intake is very important for prevention of malnutrition. The dietary macronutrients analysis is necessary for the Chinese hemodialysis patients, who have different diet style from westerners. Several valid and useful clinical tools can be used to estimate nutrients intake, one of which is the 3-day food records. In the present study, a 3-day food record was used for hemodialysis patients. However, no significant lower dietary intake was observed in hemodialysis population. A caloric intake of 30 kcal/kg/day (patients >/=60 years of age) and protein intake of 1.2 g/kg /day are recommended by K/DOQI to maintain a neutral nitrogen balance and prevent changes in body composition [19]. In our study, the mean dietary energy and protein intake was 31.9 kal/kg/d and 1.3g/kg/d, respectively. And it indicated that the major dietary macronutrients intake was sufficient in the Chinese malnourished hemodialysis patients. 

Inflammation and malnutrition are often coexisting in hemodialysis patients. It is found that 53% patients with malnutrition had signs of inflammation and 72% patients with inflammation had signs of malnutrition [20]. Our previous study and other team works also demonstrated that in malnutrition hemodialysis patients, there is a high level of inflammation [21,22]. Interestingly, in previous study, the insufficiency intake of dietary micronutrients was observed among patients with malnutrition. However, no significant differences of macronutrients (calories, protein, fat, etc) were found between normal and malnutriton groups. One possible explanation may be that we provide the basic macronutrients to maintain the patients’ physiological need. But even if the malnourished hemodialysis patients had sufficient macronutrients supplement, they had a high level of inflammation than normal nourished hemodialysis patients. It is showed that CRP, a systematic inflammatory marker, could predict malnutrition in patients with end stage renal disease. In the multivariate regression analysis of our study, we found that CRP was independently associated with MIS. However, we did not find a significant association between dietary macronutrients and MIS. That may indicate that inflammation but not dietary macronutrients insufficiency was associated with malnutrition. Or other trace elements are involved in malnutrition through promoting the inflammation development such as vitamin C [23]. Because at present, the mortality of Chinese hemodialysis patients is still very high and it does not decrease along with the living standard of Chinese population. 

There are still some limitations in this study. First, we grouped the patients by MIS, the differences of markers can be influenced by MIS to some degree. Second, we only evaluated inflammation by CRP, more serum inflammatory factors needed to be tested in the future work. Third, since several sources of error may happen during the process of the data collection e.g. errors in remembering the foods, consumed the frequency of food consumption is inaccurately reported, etc [24], so, there may exits some errors affecting results. Furthermore, because of the limitation of sample size, more large scale studies are needed to verify our findings. 

In conclusion, malnutrition is common in Chinese hemodialysis patients. In addition to traditional BMI, FM evaluation may be a relatively appropriate and better anthropometric measurement to assess the nutritional status of hemodialysis patients. Dietary protein and energy intake was generally sufficient in most hemodialysis patients. CRP, a systemic inflammatory marker, increased in the malnutrition hemodialysis patients. And CPR but not macronutrients showed a significant association with MIS which indicated that inflammtion may play a central role in the development of malnutriton. 
